# Anatomical versus Non-anatomical Resection for Hepatocellular Carcinoma with Microscope Vascular Invasion: A Propensity Score Matching Analysis

**DOI:** 10.7150/jca.32592

**Published:** 2019-07-05

**Authors:** Xiao-Ping Zhong, Yong-Fa Zhang, Jie Mei, Shao-Hua Li, Anna Kan, Liang-He Lu, Min-Shan Chen, Wei Wei, Rong-Ping Guo

**Affiliations:** 1 Department of Burn and Plastic Surgery, 2nd Affiliated Hospital of Shantou University Medical College, Shantou 515041, China;; 2 Department of Hepatobiliary Oncology of Sun Yat-sen University Cancer Center; State Key Laboratory of Oncology in South China; Collaborative Innovation Center for Cancer Medicine, Guangzhou 510060, China;; 3 Department of Hepatic Surgery, Fudan University Shanghai Cancer Center, Shanghai, China; Department of Oncology, Shanghai Medical College, Fudan University, Shanghai 200032, China.

**Keywords:** Hepatocellular carcinoma, Anatomical resection, Microscope vascular invasion, Propensity score matching, Overall survival, Disease-free survival

## Abstract

**Background:** The benefits of anatomical resection (AR) and non-anatomical resection (NAR) on hepatocellular carcinoma (HCC) patients with microscope vascular invasion (MVI) remain unknown. We aimed to investigate the prognostic outcomes of AR and NAR for HCC patients with MVI.

**Study Design:** A total of 362 consecutive HCC patients diagnosed with MVI after hepatic resection between February 2005 and December 2013 were included in this study. The patient outcomes were compared, and a 1:2 propensity score matching (PSM) analysis was applied to eliminate selection bias.

**Results:** Before PSM, compared to the NAR group, the AR group contained more patients that exceeded the Milan criteria, with larger, unilobar tumors and higher AST levels. After PSM, 100 patients were classified into the propensity-matched AR group (PS-AR), while 170 were classified into the propensity-matched NAR group (PS-NAR). Baseline data, including liver function and tumor burden measurements, were similar in the matched groups. The respective 1-, 3- and 5-year overall survival (OS) rates were 78.9%, 56.9%, and 51.5% in the PS-AR group and 76.2%, 53.0%, and 42.4% in the PS-NAR group (P = 0.301). The 1-, 3- and 5-year disease-free survival (DFS) rates were 51.1%, 44.7% and 42.0% in the PS-AR group and 44.9%, 34.3% and 26.4% in the PS-NAR group, respectively (P = 0.039). Multivariate analysis identified AR (P=0.025) as an independent favorable prognostic factor for DFS in HCC patients with MVI.

**Conclusions:** Anatomical resection was superior to non-anatomical resection for improving DFS in hepatocellular carcinoma patients with microscope vascular invasion.

## Introduction

Hepatocellular carcinoma (HCC) is the fifth most common primary malignancy and third most common cause of cancer-related mortality worldwide, especially in Asian and African countries^1^. Although many treatment options exist for HCC^2^, hepatic resection remains one of the proven potentially curative treatments^3^. High postoperative recurrence remains a serious problem, as recurrence occurs in the first 3 years after curative hepatic resection in approximately 50%-60% of cases and in the first 5 years in more than 70% of cases, even for patients with small HCCs^4^, ^5^.

Anatomical resection (AR) may be effective for the eradication of intrahepatic microscopic metastases and prevent the development of metastasis upon the systematic removal of at least one tumor-containing Couinaud's segment, while microscopic vascular dissemination is considered the main treatment route for intrahepatic metastases^6^-^8^. Many studies have demonstrated that completely eradicating tumor-bearing portal tributaries may confer better overall survival (OS) and disease-free survival (DFS) than non-anatomical resection (NAR)^9^-^12^. On the other hand, some studies indicated no differences in HCC recurrence or OS rates between the two groups subjected to curative intent resection^13^-^16^. Nevertheless, these conclusions have limited the statistical power of the data because of heterogeneities in the clinical characteristics of the patients, and no studies have compared the outcomes between AR and NAR in HCC patients with microscopic vascular invasion (MVI)^17^, ^18^. Therefore, the advantages of AR for HCC patients with MVI remain controversial.

In this retrospective study, we investigated the prognostic outcomes of AR and NAR in HCC patients with MVI. Propensity score matching (PSM)^18^, ^19^ was used to eliminate possible selection bias arising from the patients' backgrounds to better determine the impacts of the operative approaches on OS and DFS.

## Methods

### Patients

From February 2005 to December 2013, consecutive patients with HCC undergoing hepatic resection with curative intent at Sun Yat-sen University Cancer Center were evaluated for this study. All clinical data were prospectively collected in an HCC database and reviewed retrospectively.

Among these patients, only those who met all of the following inclusion criteria were selected in the study: (a) between 18 and 75 years of age with good operative tolerance, (b) had a Child-Pugh class A status, (c) had pathologically classified primary HCC, (d) had microscopic vascular invasion as determined by postoperative pathology, (e) had no anticancer treatments prior to the operation, and (f) had no evidence of extrahepatic metastasis.

Patients were excluded from the study if they met one or more of the following exclusion criteria: (a) macroscopic vascular invasion, (b) incomplete clinical data, (c) palliative hepatic resection, or (d) a history of other cancers.

### Diagnosis

All patients were diagnosed with HCC according to preoperative radiographic results and laboratory tests^20^. MVI was defined as the presence of a tumor in a portal vein, hepatic vein, or large capsular vessel of the surrounding hepatic tissue lined by endothelium that was visible only by microscopy^21^.

### Surgical procedure

Hepatic resection was performed using previously described techniques^22^, ^23^. The resection methods and resection planes were chosen preoperatively at an HCC multidisciplinary team (MDT) meeting considering the patients' background variables, including their liver function, tumor location, drainage tumor area, remnant liver volume, reserved liver function and technical difficulty of liver resection. AR was defined as the systematic removal of at least one Couinaud's segment, including left/ right hemihepatectomy, left lateral lobectomy and segmental hepatectomy. Intraoperative ultrasonography was performed if necessary to confirm the number and extent of lesions and to identify the portal or hepatic vein of the tumor-bearing section. To appropriately identify segments for removal, the liver was divided according to liver surface demarcations after finding and controlling the hepatic pedicle of the targeted part of the liver under intraoperative ultrasound guidance. NAR was defined as tumor resection with a negative tumor margin without regard to Couinaud's segments or sectional or lobar structures. The Pringle maneuver was applied if necessary.

### Propensity score matching analysis

Because this was a retrospective study and the operative approach was not assigned randomly, there was potential for confounding and selection biases between groups that could impact the comparisons of outcomes. To overcome the biases produced by disequilibrium between the two groups, PSM was conducted.

The propensity score was calculated by the logistical regression model using the following baseline characteristics as covariates: age, liver cirrhosis, prothrombin time (PT), platelets (PLT), ALB), total bilirubin (TBIL), aspartate transaminase (AST), Albumin-Bilirubin (ALBI) grade, alpha-fetoprotein (AFP), tumor extent, tumor size and Milan criteria. PSM was performed as one-to-two matching between the AR and NAR groups with nearest neighbor matching and a 0.1 caliper width using SPSS (IBM SPSS Statistics for Windows, version 19.0. IBM Corp., Armonk, NY) and Propensity Score Matching for SPSS, version 1.0 (Felix Thoemmes, Cornell University/University of Tübingen).

### Postoperative follow-up

After the liver operation, all patients were followed up by physical examination, tumor marker assessment, liver biochemistry and function assessment, blood tests, abdominal ultrasonography, and contrast-enhanced computed tomography (CT) at least every 3 months for the first year and every 6 months thereafter for more than 60 months after treatment. Complications were reported according to the Clavien-Dindo classification^24^. Recurrence was defined by findings using at least two imaging methods, such as CT and enhanced magnetic resonance imaging (MRI), and treatment for recurrent HCC was determined according to the location and number of recurrent tumors, the patient's liver function and the results of discussion among our MDT team^25^.

### Statistical analysis

The clinical database was established with SPSS. Continuous variables, presented as medians and ranges, were compared using Student's T-test or the Mann-Whitney U-test. Categorical data were compared using chi-squared and Fisher's exact tests. OS and DFS rates were determined using Kaplan-Meier survival curves and compared by the log-rank test. Prognostic factors identified as being significant in univariate analysis (P<0.1) were subjected to multivariate analysis with the Cox proportional hazards regression model. For all tests, P values less than 0.05 were considered statistically significant.

## Results

### Patient characteristics

A total of 362 patients with MVI who underwent primary resection were included in this study. The baseline characteristics of the entire study population are reported in Table 1; 124 patients undergone anatomical resection were assigned to the AR group (34.3%), and 238 undergone non-anatomical resections were assigned to the NAR group (65.7%). Compared to the NAR group, more patients in the AR group exceeded the Milan criteria before PSM, with larger, unilobar tumors and higher AST levels (Table 1). After the 1:2 PSM, 270 patients were recruited for comparison; 100 patients were classified into the propensity-matched AR group (PS-AR, 37%), and 170 were classified into the propensity-matched NAR group (PS-NAR, 63%). The background characteristics and preoperative factors of the patients in the two groups after PSM are shown in Table 1. No obvious differences were observed between the PS-AR and PS-NAR groups.

### Clinical outcomes

**Figure 1:**
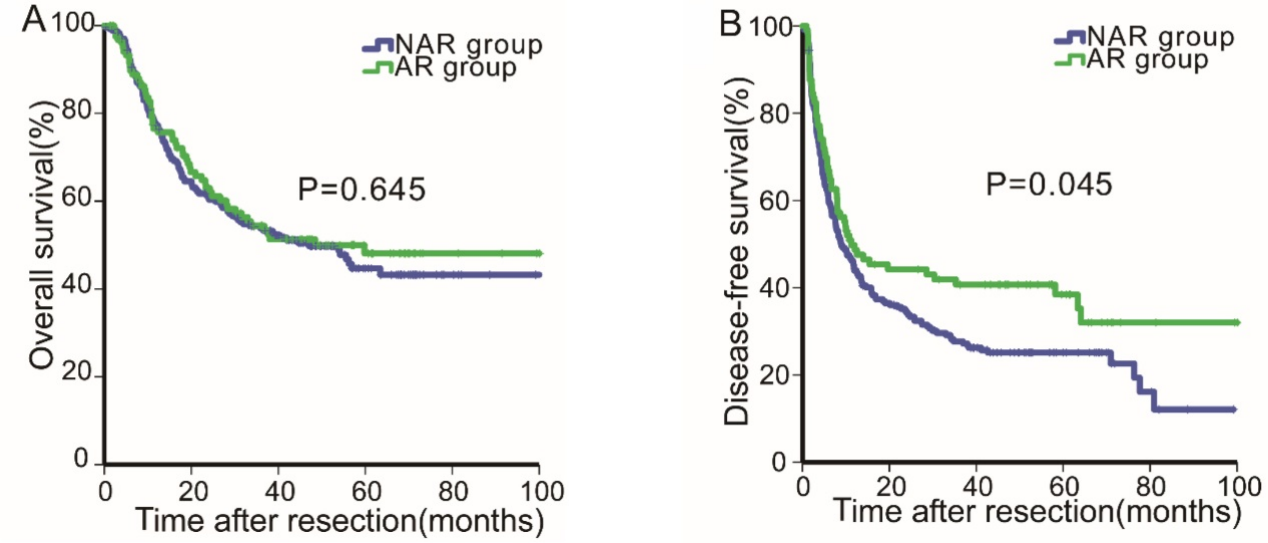
Overall survival (1A) and disease-free survival (1B) curves of patients in the anatomical

Before PSM, the median follow-up period for all patients was 30.39 months (range, 0.2-108.0 months). For all patients, the 1-, 3- and 5-year OS rates were 77.0%, 54.0% and 46.0%, respectively, and the 1-, 2- and 3-year DFS rates were 45.6%, 37.1% and 31.4%, respectively. Next, the OS and DFS curves for the two groups were stratified according to the surgical procedure employed (Fig. 1A & 1B). The DFS rates of the AR group (1-, 2- and 3-year: 49.8%, 44.2%, 40.7%, respectively) were better than those of the NAR group (1-, 2- and 3-year: 44.0%, 34.2%, 27.7%, respectively, P=0.045, Fig. 1B). By contrast, the OS rates of the AR group were not statistically different from those of the NAR group (AR vs. NAR: 76.5%, 54.4%, 48.1% vs. 77.2%, 53.8%, 44.7%, respectively, P=0.645, Fig. 1A).

**Figure 2:**
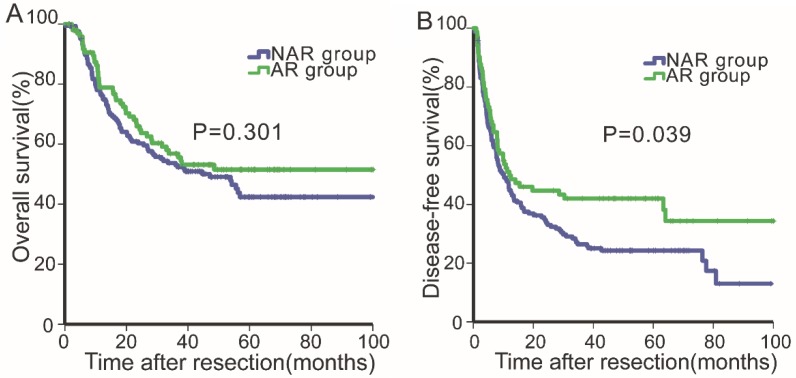
Overall survival (2A) and disease-free survival (2B) curves of patients in the anatomical resection and non-anatomical resection groups after propensity score matching analysis.

After PSM, the median follow-up period was 31.46 months (range, 0.23-136.74 months). The 1-, 3- and 5-year OS rates were 77.2%, 54.5 and 46.1%, respectively, and the 1-, 2- and 3-year DFS rates were 46.5%, 37.7% and 31.9%, respectively. The OS and DFS curves for the two groups (PS-AR vs. PS-NAR) were stratified according to the surgical procedure employed (Fig.2A & 2B). For the PS-AR group, the respective 1-, 3- and 5-year OS rates were 78.9%, 56.9%, and 51.5%, and the median survival time was 33.4 months. For the PS-NAR group, the respective 1-, 3-, and 5-year OS rates were 76.2%, 53.0%, and 42.4%, and the median survival time was 30.6 months (P=0.301, Fig. 2A). Significant differences existed between the DFS rates of the PS-AR and PS-NAR groups (P=0.039, Fig. 2B). The 1-, 2- and 3- DFS rates and median DFS time were 51.1%, 44.7%, 42.0%, and 12.0 months, respectively, for the PS-AR group and 44.9%, 34.3%, 26.4% and 10.1 months, respectively, for the PS-NAR group.

### Surgical variables and postoperative complications

Table 2 lists the surgery-related variables, tumor variables, postoperative complications and types of recurrence between the AR and NAR groups. Although the patients who underwent AR showed a greater portion of major resection (39% vs. 18.8%, P=0.000) and a longer operation time (186.7±63.0 min vs. 171.8±50.8 min, P=0.043) than those who underwent NAR, the postoperative complications (P=0.499), blood loss (552.3±1076.1 ml vs. 457.6±452.7 ml, P=0.315) and time of the Pringle maneuver (10.5±13.2 vs. 14.0±11.2, P=0.060) were not significantly different between the two group before and after PSM.

### Univariate and multivariate analyses of overall survival and disease-free survival

Univariate and multivariate analyses were performed to identify the predictors that influenced OS and DFS both before and after PSM, and the results are shown in Tables 3 and 4. Before matching, univariate analysis revealed that the PT, AST, ALBI grade, Milan criteria, AFP, tumor extent, tumor number and tumor size factors influenced the OS rates, while multivariate analysis identified PT (P=0.03), AST (P=0.034), TBIL (P=0.022) and tumor number (P=0.001) as independent risk factors. Additionally, the PT, AST, ALBI grade, Milan criteria, AFP, tumor extent, tumor number, tumor size and AR factors affected the DFS rates according to univariate analysis, and multiple analysis indicated that PT (P=0.008), AFP (P=0.020), multiple tumors (P=0.003) and AR (P=0.009) were independent prognostic factors for DFS.

Table 4 shows the factors (PT, AST, liver cirrhosis, ALBI grade, Milan criteria, tumor extent, tumor number and tumor size) that influenced the OS rates of MVI patients after PSM. These analyses also indicated that the DFS rates were significantly related to the PT, serum album, ALBI grade, Milan criteria, tumor extent, tumor number, tumor size and AR factors. Multivariate analysis identified that PT (P=0.001) and multiple tumors (P=0.001) independently influenced the OS rates. PT (P=0.000), album (P=0.031), AFP (0.008), bilobar tumor extent (P=0.004), multiple tumors (P=0.019) and AR (P=0.025) were independent unfavorable prognostic factors for DFS in HCC patients.

## Discussion

In this retrospective study, the clinical outcomes of AR and NAR performed with curative intent on HCC patients with MVI were investigated using PSM analysis. Furthermore, our study indicated that AR performed on HCC patients with MVI provided better DFS rates after initial resection. Univariate and multivariate analyses indicated that AR was an independent favorable prognostic factor for DFS.

HCC with MVI has a high incidence of recurrence and metastasis via the intrahepatic portal vein system, which is the main reason for its bad prognosis,^26^, ^27^ and MVI has been reported as a risk survival factor following resection. Survival chances are improved by eliminating macroscopic and microscopic liver metastases and preserving functional liver parenchymal cells to the greatest extent possible. Therefore, AR is a theoretically ideal procedure, as it may remove the portal tributaries bearing the tumor completely and reduce the ischemic operation area^28^, ^29^. In clinical practice, surgeons often choose AR for treating patients with good liver function, small tumors and no cirrhosis to reduce the risks in terms of difficulty of the AR technique and remnant liver functions^19^, ^30^. Indeed, our study showed significantly longer operation times for the AR procedure. The postoperative complications, intraoperative blood loss and rates of homologous blood transfusion were similar between the AR and NAR groups.

Previous studies compared the clinical outcomes of HCC patients subjected to the AR and NAR procedures^17^, ^31^, ^32^. Zhou et al.^11^, Eguchi et al.^12^ and Hasegawa et al.^33^ concluded that AR was superior to NAR for prolonging OS and DFS after hepatic resection. However, other researchers reported different findings^16^, ^31^. Marubashi et al.^18^ proposed that the OS and DFS rates in the AR and NAR groups were not statistically different. Chen et al.^30^ and Zhao et al.^17^ reported that AR contributed to better DFS rates, but the OS rates between the AR and NAR groups were similar. Whether AR is superior to NAR remains controversial, and data on the effects of performing AR on HCC patients with MVI are lacking. This study confirmed that AR resulted in better DFS rates than NAR. Though the Kaplan-Meier survival curves of OS were not significantly different between the AR and NAR groups, the 5-year survival rates in the AR group were higher than those in the NAR group both before and after PSM analysis (48.1% vs. 44.7% and 51.5% vs. 42.4%, respectively).

In the original unmatched group, the short-term OS rates were similar between patients undergoing AR and NAR (1-, 3-year: 76.5%, 54.4% vs. 77.2%, 53.8%, respectively). The AR group included a higher proportion of patients with large tumors, multiple tumors, liver cirrhosis and high AFP levels than the NAR group. These were all known risk factors associated with tumor recurrence and reduced survival, which might explain why the short-term outcomes were nearly the same between the two groups^34^, ^35^. After using 1:2 PSM analysis to reduce selection bias, the 1-, 3- and 5-year OS rates in the PS-AR group were higher than those in the PS-NAR group (78.9%, 56.9%, and 51.5% vs. 76.2%, 53.0%, and 42. 4%, respectively). When comparing AR and NAR patients, the differences in the long-term survival rates were more deviated than those in the short-term survival rates. In summary, AR patients have better long-term survival prognoses than NAR patients.

In this retrospective study, a 1:2 propensity score matching (PSM) analysis was used to eliminate possible selection biases between two groups. As a result, selection biases were significantly reduced but not completely eliminated which could impact the clinical outcomes. In the AR cohort, some patients underwent hemihepatectomy with multiple tumors (26.6% vs. 24.8%) and larger tumor sizes (8.1±3.7 cm vs. 6.6±3.7 cm, P<0.000). Although PSM was conducted, the disequilibrium between groups still existed (multiple tumor 25.0% vs. 18.8%, tumor sizes 7.8±3.7 cm vs. 7.1±3.6 cm), which might have impacts on the clinical outcomes. However, anatomical resection may systematic removed microscopic metastases and prevent metastasis. Therefore, the OS of AR-group did not find significant advantage, but DFS was significantly better than that of control group, and the 5-year OS rates was better (51.5% vs. 42. 4%). In conclusion, anatomical hepatectomy may lead to better clinical outcomes for HCC patients with MVI.

This study did have several limitations. First, the research was designed as a single-center, retrospective, and non-randomized controlled study. The operative procedures were decided upon by various clinical doctors in the MDT group. Even if PSM was used to limit the number of similar baseline characteristics, possible selection biases between the groups remained. Therefore, multicenter, randomized controlled trials are required to confirm the role of AR in HCC patients with MVI.

In conclusion, our study indicated that AR improved the RFS rates in patients with MVI. If a surgical procedure is feasible and preoperative examination shows the possibility of MVI, AR is recommended.

## Conclusions

In the present study, we aimed to investigate the prognostic outcomes of AR and NAR for HCC patients with MVI. PSM was used to eliminate possible selection bias arising from the patients' backgrounds to better determine the impacts of the operative approaches on OS and DFS.

As a result, we found that AR was superior to NAR for improving DFS in HCC patients with MVI. Indeed, the 5-year survival rates in the AR group were higher than those in the NAR group.

## Figures and Tables

**Table 1:** Background characteristics of patients before and after propensity score matching analysis

Characteristics	Before PSM			After PSM		
	AR (n=124)	NAR (n=238)	P-value	PS-AR (n=100)	PS-NAR (n=170)	P-value
**Epidemiology**						
Age (year)	46±11.5	47.9±11.4	0.140	46.9±11.5	48.1±11.2	0.420
Gender			0.537			0.311
Male	110(88.7)	216(90.8)		88(88.0)	156(91.8)	
Female	14(11.3)	22(9.2)		12(12.0)	14(8.2)	
**Etiology**						
Virus(HBV/HCV)	109(87.9)	224(94.1)	0.417	87(87.0)	158(92.9)	0.697
Others	15(12.1)	14(5.9)	0.039	13(13.0)	12(7.1)	0.104
**Liver function**						
PT	12.2±1.1	12.2±1.1	0.75	12.1±1.2	12.2±1.1	0.363
PLT (*109/L)	193.6±72.4	190.9±72.8	0.736	195.7±73.6	185.9±70.9	0.283
ALB (g/L)			0.745			0.751
>35	119(96.0)	230(96.6)		97(97.0)	162(95.3)	
≤35	5(4.0)	8(3.4)		3(3.0)	8(4.7)	
AST (U/L)			0.015			0.175
>45	59(47.6)	82(34.5)		43(43.0)	59(34.7)	
≤45	65(52.4)	156(65.5)		57(57.0)	111(65.3)	
TBIL (umol/L)			0.160			0.513
>17.5	41(33.1)	62(26.1)		32(32.0)	48(28.2)	
≤17.5	83(66.9)	176(73.9)		68(68.0)	122(71.8)	
Cirrhosis	91(73.4)	172(72.3)	0.821	74(74.0)	125(73.5)	0.932
ALBI			0.887			0.378
Grade 1	91(73.4)	173(72.7)		75(75.0)	119(70.0)	
Grade 2&3	33(26.6)	65(27.3)		25(25.0)	51(30.0)	
Milan	29(23.4)	84(35.3)	0.020	27(27.0)	54(31.8)	0.409
**Tumor burden**						
AFP (ng/ml)			0.595			0.688
>400	62(50.0)	112(47.1)		49(49.0)	79(46.5)	
≤400	62(50.0)	126(52.9)		51(51.0)	91(53.5)	
Tumor extent			0.010			0.629
Unilobar	122(98.4)	218(91.6)		98(98.0)	168(98.8)	
Bilobar	2(1.6)	20(8.4)		2(2.0)	2(1.2)	
Tumor number			0.705			0.230
Single	91(73.4)	179(75.2)		75(75.0)	138(81.2)	
Multiple	33(26.6)	59(24.8)		25(25.0)	32(18.8)	
Tumor size (cm)			0.031			0.326
>5	94(75.8)	154(64.7)		75(75.0)	118(69.4)	
≤5	30(24.2)	84(35.3)		25(25.0)	52(30.6)	

Values are expressed as the mean ± SD or no. (%), unless otherwise indicated; PSM, propensity score matching; AR, anatomical resection; NAR, non-anatomical resection; PS-AR, propensity-matched anatomical resection; PS-NAR, propensity-matched non-anatomical resection; HBV, hepatitis B virus; HCV, hepatitis C virus; PT, prothrombin time; PLT, platelet count; ALB albumin; AST, aspartate aminotransferase; TBIL, total bilirubin; ALBI, Albumin-Bilirubin grade; Milan, within Milan criteria; AFP, alpha-fetoprotein.

**Table 2:** Surgery-related characteristics and postoperative outcome

Characteristics	Before PSM			After PSM		
	AR (n=124)	NAR (n=238)	P-value	PS-AR (n=100)	PS-NAR (n=170)	P-value
**Surgical variables**						
Surgical margin (≥1cm)	77 (62.1)	125 (52.5)	0.082	64 (64)	91 (53.5)	0.093
Scope of resection			0.000			0.000
Major resection	51 (41.1)	52 (21.8)		39 (39)	32 (18.8)	
Minor resection	73 (58.9)	186 (78.2)		61 (61)	138 (81.2)	
Operation time (min)	191.4±65.1	170.3±47.6	0.002	186.7±63.0	171.8±50.8	0.043
Time of Pringle's maneuver	10.4±13.3	13.8±10.8	0.015	10.5±13.2	14.0±11.2	0.060
Blood loss (ml)	568.0±1001.3	437.8±430.3	0.169	552.3±1076.1	457.6±452.7	0.315
**Tumor variables**						
Single tumor	91(73.4)	179(75.2)	0.705	75(75.0)	138(81.2)	0.230
Tumor size>5(cm)	94(75.8)	154(64.7)	0.031	75(75.0)	118(69.4)	0.326
Capsule (with)	83 (66.9)	153 (64.3)	0.615	67 (67)	117 (68.8)	0.756
Edmondson grades^#^			0.248			0.183
I, II	75 (60.5)	159 (66.8)		62 (62.0)	119 (70.0)	
III, IV	49 (39.5)	79 (33.2)		38 (38.0)	51 (30.0)	
Complications^*^			0.279			0.499
Ⅰ	60 (48.4)	119 (50.0)		39 (39.0)	59 (34.7)	
Ⅱ	32 (25.8)	75 (31.5)		25 (25.0)	52 (30.6)	
Ⅲ	27 (21.8)	40 (16.8)		31 (31.0)	55 (32.4)	
Ⅳ	5 (4.0)	4 (1.7)		5 (5.0)	4 (2.4)	
Ⅴ	0	0		0	0	
**Recurrence**						
Intrahepatic recurrence	65 (52.4)	123 (51.7)	0.498	51 (51.0)	87 (51.2)	0.978
Extrahepatic metastasis	32 (25.8)	54 (22.7)	0.508	24 (24.0)	45 (26.5)	0.653
Vascular invasion	13 (10.5)	21 (8.8)	0.607	10 (10.0)	18 (10.6)	0.878

Values are expressed as the mean ± SD or no. (%), unless otherwise indicated; PSM, propensity score matching; AR, anatomical resection; NAR, non-anatomical resection; PS-AR, propensity-matched anatomical resection; PS-NAR, propensity-matched non-anatomical resection; ^#^ According to the Edmondson and Steiner grading system; ^*^According to Clavien-Dindo classification of surgical complications.

**Table 3:** Univariate analysis and multivariate analysis of overall survival and disease-free survival for patients before propensity matching analysis.

Variables	OS				DFS			
	Univariate analysis	Multivariate analysis			Univariate analysis	Multivariate analysis		
	P-value	HR	95%CI	P-value	P-value	HR	95%CI	P-value
Age (≤50/>50) (year)	0.832				0.643			
Gender (male/female)	0.247				0.528			
Virus ( positive/negative)	0.475				0.176			
PT (≤13.5/>13.5) (sec)	0.000	1.976	1.262-3.094	0.03	0.001	1.756	1.160-2.659	0.008
PLT (≤100/>100) (*109/L)	0.924				0.816			
ALB (≤35/>35) (g/L)	0.217				0.234			
AST (≤45/>45) (U/L)	0.000	1.420	1.027-1.965	0.034	0.048			
TBIL (≤17.5/>17.5) (umol/L)	0.062	1.494	1.059-2.110	0.022	0.329			
Cirrhosis (yes/no)	0.211				0.557			
ALBI ( grade 1/grade 2&3)	0.020				0.024			
Milan	0.000				0.000			
AFP (≤400/>400) (ng/ml)	0.036				0.011	1.367	1.051-1.778	0.020
Tumor extent (unilobar/bilobar)	0.023				0.047			
Tumor number (single/multiple)	0.000	1.809	1.274-2.568	0.001	0.000	1.592	1.166-2.174	0.003
Tumor size (≤5/>5) (cm)	0.000				0.000			
Operative procedure (AR/NAR)	0.645				0.045	1.504	1.105-2.045	0.009

OS, overall survival; DFS, disease-free survival; HR, hazard ratio; CI, confidence interval; AR, anatomical resection; NAR, non-anatomical resection; PT, prothrombin time; PLT, platelet count; ALB, albumin; AST, aspartate aminotransferase; TBIL, total bilirubin; ALBI, Albumin-Bilirubin grade; Milan, within Milan criteria; AFP, alpha-fetoprotein.

**Table 4:** Univariate analysis and multivariate analysis of overall survival and disease-free survival for patients after propensity matching analysis.

Variables	OS				DFS			
	Univariate analysis	Multivariate analysis			Univariate analysis	Multivariate analysis		
	P-value	HR	95%CI	P-value	P-value	HR	95%CI	P-value
Age (≤50/>50) (year)	0.914				0.243			
Gender (male/female)	0.252				0.923			
Virus ( positive/negative)	0.904				0.487			
PT (≤13.5/>13.5) (sec)	0.000	2.475	1.475-4.153	0.001	0.001	2.410	1.486-3.909	0.000
PLT (≤100/>100) (*109/L)	0.899				0.996			
ALB (≤35/>35) (g/L)	0.182				0.041	2.224	1.076-4.595	0.031
AST (≤45/>45) (U/L)	0.018				0.176			
TBIL (≤17.5/>17.5) (umol/L)	0.217				0.766			
Cirrhosis (yes/no)	0.043				0.534			
ALBI ( grade 1/grade 2&3)	0.096				0.081			
Milan	0.003				0.000			
AFP (≤400/>400) (ng/ml)	0.123				0.031	1.510	1.113-2.050	0.008
Tumor extent (unilobar/bilobar)	0.021				0.000	5.963	1.762-20.174	0.004
Tumor number (single/multiple)	0.000	1.976	1.323-2.951	0.001	0.003	1.560	1.076-2.262	0.019
Tumor size (≤5/>5) (cm)	0.004				0.000			
Operative procedure (AR/NAR)	0.301				0.039	1.477	1.049-2.079	0.025

OS, overall survival; DFS, disease-free survival; HR, hazard ratio; CI, confidence interval; AR, anatomical resection; NAR, non-anatomical resection; PT, prothrombin time; PLT, platelet count; Alb, albumin; AST, aspartate aminotransferase; TBIL, total bilirubin; ALBI, Albumin-Bilirubin grade; Milan, within Milan criteria; AFP, alpha-fetoprotein.
